# An Automated Approach for the Detection of Alzheimer's Disease From Resting State Electroencephalography

**DOI:** 10.3389/fninf.2022.924547

**Published:** 2022-07-11

**Authors:** Eduardo Perez-Valero, Christian Morillas, Miguel A. Lopez-Gordo, Ismael Carrera-Muñoz, Samuel López-Alcalde, Rosa M. Vílchez-Carrillo

**Affiliations:** ^1^Department of Computers Architecture and Technology, University of Granada, Granada, Spain; ^2^Brain Computer Interface Laboratory, Research Center for Information and Communications Technologies, University of Granada, Granada, Spain; ^3^Department of Signal Theory, Telematics, and Communications, University of Granada, Granada, Spain; ^4^Cognitive Neurology Group, Hospital Universitario Virgen de las Nieves, Granada, Spain; ^5^Hospital Universitario San Rafael, Granada, Spain

**Keywords:** Alzheimer's disease, EEG, machine learning, disease detection, classification

## Abstract

Early detection is crucial to control the progression of Alzheimer's disease and to postpone intellectual decline. Most current detection techniques are costly, inaccessible, or invasive. Furthermore, they require laborious analysis, what delays the start of medical treatment. To overcome this, researchers have recently investigated AD detection based on electroencephalography, a non-invasive neurophysiology technique, and machine learning algorithms. However, these approaches typically rely on manual procedures such as visual inspection, that requires additional personnel for the analysis, or on cumbersome EEG acquisition systems. In this paper, we performed a preliminary evaluation of a fully-automated approach for AD detection based on a commercial EEG acquisition system and an automated classification pipeline. For this purpose, we recorded the resting state brain activity of 26 participants from three groups: mild AD, mild cognitive impairment (MCI-non-AD), and healthy controls. First, we applied automated data-driven algorithms to reject EEG artifacts. Then, we obtained spectral, complexity, and entropy features from the preprocessed EEG segments. Finally, we assessed two binary classification problems: mild AD vs. controls, and MCI-non-AD vs. controls, through leave-one-subject-out cross-validation. The preliminary results that we obtained are comparable to the best reported in literature, what suggests that AD detection could be automatically detected through automated processing and commercial EEG systems. This is promising, since it may potentially contribute to reducing costs related to AD screening, and to shortening detection times, what may help to advance medical treatment.

## 1. Introduction

Alzheimer's disease (AD) is a neurogenerative disease that represents 60–70% of the dementia cases (Amezquita-Sanchez et al., 2019). According to the 2018 World Alzheimer's Report, more than fifty million people suffer from dementia and this prevalence is likely to triple by 2050 (Patterson, 2018). AD patients experience a progressive decline in multiple cognitive areas, such as memory, orientation, and reasoning, to the extent of interfering with their daily-life activities. Although this disease was initially documented in 1906, its etiology is still uncertain, and a final diagnosis can only be performed at brain autopsy. However, according to the literature, two major hallmarks begin to form before the impairment is noticeable: amyloid plaques and neurofibrillary tangles (Serrano-Pozo et al., 2011). Amyloid plaques are deposits of proteins that lose their normal structure and accumulate around the neurons. Likewise, neurofibrillary tangles are thickened fibrils that surround the neuron nucleus. In this context, mild cognitive impairment (MCI) is often referred as a condition between normal aging and AD (Petersen et al., 2001). MCI patients experience unexpected memory losses for age but these do not interfere with their daily-life activities. Nonetheless, MCI patients transition to AD at a faster rate than healthy individuals of the same age. Although presently there is no cure for AD, early detection can help control the progression of the disease and postpone intellectual decline. This, alongside the high prevalence of the disease, evidence the need for non-invasive accessible detection techniques.

The purpose of AD detection techniques is to reveal the physical and cognitive symptoms linked to the disease. Traditionally, this is accomplished through neuropsychological tests and medical procedures. Neuropsychological tests are designed to evaluate cognitive areas affected early in the AD course, such as memory, language, and orientation (Carnero-Pardo et al., 2007; Ciesielska et al., 2016; Matias-Guiu et al., 2017). Although neuropsychological tests are extensively applied, previous works have evidenced their lack of sensitivity and high variability (Mendiondo et al., 2000; Kuslansky et al., 2004). Conversely, medical procedures aim to unfold the damages produced to specific brain structures. Among these procedures, cerebrospinal fluid (CSF) analysis is the most reliable. However, this fluid is extracted *via* lumbar puncture, an invasive medical procedure with reported side effects (Virhammar et al., 2012). Alternatively, medical imaging techniques are designed to render functional or static images of the brain. The most extended techniques include: magnetic resonance imaging (MRI) (Farooq et al., 2017; Hojjati et al., 2017), positron emission tomography (PET) (Li et al., 2015; Lu et al., 2018), and single photon emission computed tomography (SPECT) (Gorriz et al., 2011; Bi and Wang, 2019). Although these techniques yield accurate results, they present some drawbacks: they usually involve long waiting lists, their analysis is often based on visual inspection, and some of them require invasive procedures.

In this context, for the past decade researchers have studied the role of electroencephalography (EEG) for AD detection. EEG is a neurophysiology technique to measure the electrical activity of the brain through electrodes placed on the scalp. EEG is portable, non-invasive, and affordable compared to medical imaging procedures. Consequently, it represents a promising approach for the detection of neurological diseases. Indeed, researchers have recently combined EEG signal processing and machine learning algorithms to discriminate AD and MCI patients from age matched controls (Trambaiolli et al., 2011, 2017; Aghajani et al., 2013; McBride et al., 2013; Wang et al., 2015; Kashefpoor et al., 2016; Cassani et al., 2017; Fiscon et al., 2018; Ruiz-Gomez et al., 2018; Durongbhan et al., 2019; Khatun et al., 2019; Ieracitano et al., 2020; Perez-Valero et al., 2022). These works typically analyze the EEG signals in terms of spectral content, complexity, and synchronization, since previous studies have found these features are affected in AD patients. For instance, in Gallego-Jutgla et al. (2015), the authors classified AD patients and controls based on frequency and power features using linear discriminant analysis. Likewise, in Wang et al. (2015), researchers performed cluster analysis on power spectral density (PSD) and coherence features to differentiate AD patients and controls. Additionally, the authors of McBride et al. (2015) and McBride et al. (2013) implemented a support vector machine (SVM) to classify early stage AD and MCI patients based on coherence and entropy measures, respectively.

These works demonstrate the scientific community has made unquestionable progress toward EEG-based AD detection. Furthermore, recent advances in computational neuroscience have provided new insights about the collective behavior of the brain by attempting to explain the mechanisms driving the interaction between neuronal and synaptic processes, what may contribute to deepening our understanding of how neurodegeneration evolves over the course of AD (Caligiore et al., 2020; Jones et al., 2022). Indeed, in recent studies, researchers endeavor to bridge the gap in efficiency and cognitive skills between actual models and their biological equivalents (Yang et al., 2022a,b). In this context, to understand the complexity of the human brain, models are required to account for the morphological features responsible for neural dynamics and the high-level scale of the human neural network. For this purpose, approaches such as Yang et al. (2020), based on a reduced compartmental model, have been proposed recently. Furthermore, to implement large-scale neuromorphic systems, online learning, and fault-tolerant operation is mandatory (Vu et al., 2019; Yang et al., 2021). With regard to actual AD detection approaches, there are still methodological decisions that hinder the application of automated detection models. This includes the use of clinical EEG acquisition systems, EEG artifact cleaning through visual inspection and annotations, and complex electrode montages. To tackle this, in this paper, we wanted to perform a preliminary evaluation of a automated EEG classification approach for AD. To simplify the electrode setup, we used a commercial EEG acquisition device with only sixteen electrodes. Furthermore, to automatically process the acquired signals, we applied the Autoreject algorithm and independent component analysis (ICA). Then, we extracted spectral and complexity features from all the EEG channels. Finally, to assess the general performance of our model, we implemented leave-one-subject-out (LOSO) cross-validation. In order to evaluate our approach, we conducted a preliminary study in collaboration with the Cognitive and Behavioral Neurology Unit (CBNU) at Hospital Universitario Virgen de las Nieves de Granada (Spain) to discriminate MCI-non-AD patients, mild AD patients, and healthy controls. Although the sample size that we studied is reduced, the results that we obtained are promising, as our automated approach yielded classification results that are comparable to the best in literature. We believe the results derived from this work may contribute to opening the door to forthcoming ubiquitous, affordable, and accurate detection of mild AD and MCI-non-AD, nonetheless, further studies must validate the conclusions drawn in this study.

This paper is structured as follows: first, we report the methods and materials that we employed throughout the study; then, we describe the results that we obtained; subsequently, we discuss these results, and we compare our approach with analogous studies; finally, we draw conclusions, evaluate the impact and limitations of this research, and provide some guidelines for future studies.

## 2. Materials and Methods

Throughout the following subsections, we describe the main aspects of the study methodology, including participants, experimental procedure and setup, EEG signal processing, feature extraction, and classification.

### 2.1. Participants

Twenty-six volunteers participated in the study. However, due to poor signal quality (participants 21 and 26), lack of cooperation (participants 9 and 18), and presence of a condition that may have impacted the analysis (participant 12), we excluded the data from five participants. Consequently, we only studied the recordings from 21 participants. The head of the CBNU at Hospital Universitario Virgen de las Nieves de Granada recruited the participants the week before the experiment. Table 1 displays the group, age, and sex distributions of the participants. Thirteen of the participants were patients of the neurology unit at Hospital Universitario Virgen de las Nieves. Due to memory complaints, all these patients had undergone one of the following two medical trials during the past year: (a) measurement of Aβ42, p-tau, and total tau in CSF, or (b) β-amyloid PET (florbetaben PET). We cataloged the participants whose medical trials yielded positive biomarkers as mild AD, otherwise, we cataloged them as MCI-non-AD. The remaining participants were healthy controls. We conducted the study following a protocol approved by the local ethics committee at Hospital Universitario Virgen de las Nieves de Granada. Additionally, the participants signed an informed consent before the experiment onset, and CBNU personnel monitored them throughout the study session.

**Table 1:** Group, sex, and age distributions of the participants engaged for this study.

**Group**	**Females**	**Males**	**Age**
NC	7	1	67 ± 3.5
MCI-non-AD	0	5	73.4 ± 7.1
Mild AD	5	3	68.8 ± 4.9

*The values reported in the Age column represent the mean ± the standard deviation*.

### 2.2. Experimental Procedure and Setup

First, we asked the participants to read and sign the informed consent. Then, we briefed them on the study details and the basics of EEG acquisition. It is worth to note that the participants of this study were recruited with two objectives in mind: (1) go through a cognitive test, and (2) participate in the study reported in this paper. The cognitive test consisted of two tasks of approximately 10 min whose details are out of the scope of this paper. In parallel, we recorded 3 min of the eye-open resting state activity of the participants in three key moments: before the first task, after the first task, and after the second task. To avoid edge effects, we only considered the central 2-min window of each recording. For the analysis reported in this paper, we concatenated the three recordings, thus, we ended up with an effective 6-min EEG recording per participant.

**Figure 1:**
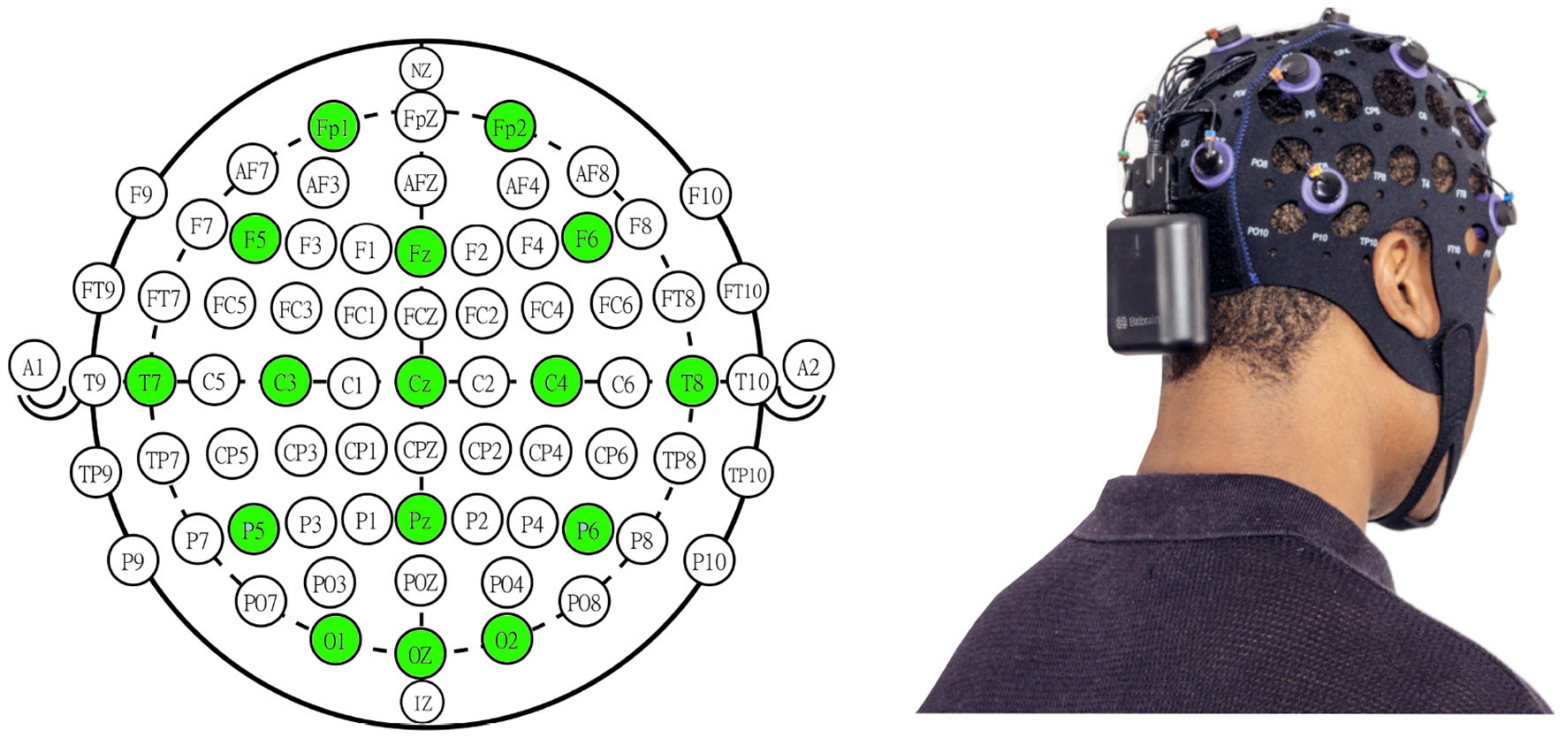
**(Left)** Sixteen-channel montage used for the present study (in green) in the extended 10-20 International System. We selected this montage to evenly cover the scalp area. **(Right)** Versatile semi-dry EEG acquisition system utilized for the data capture. The system includes a Bluetooth acquisition module placed below the occipital area and an EEG headset with semi-dry electrodes. Please, note that the right panel image does not represent the actual montage used in this study and it was included to illustrate the acquisition system and headset.

For the acquisition, we used the Versatile system by Bitbrain (Zaragoza, Spain), a commercial wearable device consisting of an EEG cap with semi-dry electrodes and a Bluetooth acquisition module that works at a fixed sampling rate of 256 Hz. This sampling rate has been previously used in related studies (Garn et al., 2017; Gouw et al., 2017; Amezquita-Sanchez et al., 2019; Ieracitano et al., 2019). For the electrode montage, we selected sixteen channels located at positions Fp1, Fp2, F5, Fz, F6, T7, T8, C3, Cz, C4, P5, Pz, P6, O1, Oz, and O2 of the extended 10-20 International System, and we referenced them to the left ear lobe. We used this setup to uniformly cover the scalp according to previous AD detection studies (Wang et al., 2015; Kulkarni and Bairagi, 2017; Fiscon et al., 2018). Figure 1 shows the electrode montage selected for this study alongside the Versatile EEG system.

### 2.3. Signal Preprocessing

We applied the procedure described in this subsection to the 6-min EEG recordings of the participants. First, we filtered the raw EEG using a 1690 order FIR filter with 1–45 Hz bandpass and zero phase-shift. We used a FIR filter instead of an IIR filter because our study did not involve high throughput constraints (Ifeachor and Jervis, 2002), and we prioritized filter stability and control. Then, we segmented the filtered EEG into 4-s epochs without overlapping. According to the systematic review on resting state EEG for AD diagnosis by Cassani et al. (2018), this epoch length lays within one of the most common epoch length ranges considered in literature (see Table 17 in the aforementioned review article).

**Figure 2:**
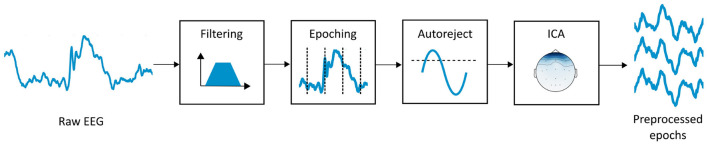
EEG signal processing pipeline. First, we applied a FIR filter with bandpass 1–45 Hz to remove the power line interference and retain the spectral content in the desired frequency range. Then, we segmented the filtered EEG into 4-s epochs without overlapping. Finally, we performed automated artifact removal through Autoreject algorithm and ICA.

Regarding artifact rejection, in order to keep our approach automated, we applied the Autoreject algorithm. Autoreject is a data-driven artifact rejection algorithm that combines cross-validation and Bayesian optimization to find a separate threshold per channel. It also marks bad trials when most channels display high-amplitude artifacts, and allows channel interpolation. Therefore, bad data segments can be repaired instead of discarded. In terms of performance, Autoreject has been validated against multiple datasets, and reportedly performed equally or better than common artifact processing approaches. We refer the interested reader to Jas et al. (2017), where the developers of the algorithm provide an exhaustive interpretation of this validation. Additionally, we also implemented blink artifact rejection through independent component analysis (ICA). ICA enables the decomposition of a signal made from the combination of multiple sources into those sources and a mixing matrix (Schlink et al., 2017; Echtioui et al., 2020). Since EEG represents the combination of internal neurological sources, ICA is usually applied to identify and remove artifactual components such as blinks. Blink components display high variance and present a spatial distribution toward the frontotemporal region of the head (Shahbakhti et al., 2022). Therefore, to identify them, we followed an automated electro-oculogram proxy approach. To this end, we determined the ICA component whose correlation with Fp1 time series was the highest, and we reconstructed the EEG without that component. Figure 2 represents the signal processing pipeline described in this subsection.

### 2.4. Feature Extraction

After preprocessing, we used MNE Python toolbox to extract three features per channel from each of the preprocessed 4-s EEG epochs: relative power (RP) in the five main EEG bands, Hjorth complexity (HC), and spectral entropy (SE). Consequently, we extracted a total of 112 features (80 for the RP, 16 for the HC, and 16 for the SE) per epoch. We considered these features since they have been already applied in analogous studies (Poil et al., 2013; McBride et al., 2014; Wang et al., 2015; Trambaiolli et al., 2017; Houmani et al., 2018). RP represents the fraction of the total power of the signal that is contained in a particular frequency band. To estimate this parameter, we applied the default MNE Python settings, that include the use of Welch's method for the calculation of the PSD. On the other hand, HC is one of the three Hjorth parameters (activity, mobility, and complexity), and is calculated as the ratio between the mobility of the first derivative of the signal and the mobility of the signal itself (Paivinen et al., 2005). Finally, SE is defined as the Shannon entropy of the power spectrum of the signal. SE represents the uniformity of the power spectrum distribution, and, hence, the irregularity of the EEG. SE is minimal for a pure sine wave and maximal for white noise (Inouye et al., 1991). The formulas for the extracted features are presented in Equations (1)–(3).


(1) $$\begin{array}{r} RP=\frac{\overset{f_{o}}{\underset{f_{i}}{\sum }}P}{\underset{\forall f}{\sum }P} \end{array}$$

(2) $$\begin{array}{r} HC=\frac{\sigma_{s^{\prime \prime }}/\sigma_{s^{\prime }}}{\sigma_{s^{\prime }}/\sigma_{s}} \end{array}$$

(3) $$\begin{array}{r} SE=-\underset{f}{\sum }S\left(f\right)*\log_{2}S\left(f\right) \end{array}$$

In Equation (1), *f*_*i*_ and *f*_*o*_ represent the lower and upper frequencies of an EEG band, and the denominator represents the total power of the EEG signal. We estimated RP for the main EEG frequency bands: delta (1–4 Hz), theta (4–8 Hz), alpha (8–13 Hz), beta (13–30 Hz), and gamma (>30 Hz). In Equation (2), σ_*s*_, $\sigma_{s^{\prime }}$, and $\sigma_{s^{\prime \prime }}$ represent the standard deviations of the signal epoch under analysis, of its first, and of its second derivative, respectively. Finally, in Equation (3), *f* represents frequency, and *S* represents the normalized power spectrum.

**Figure 3:**
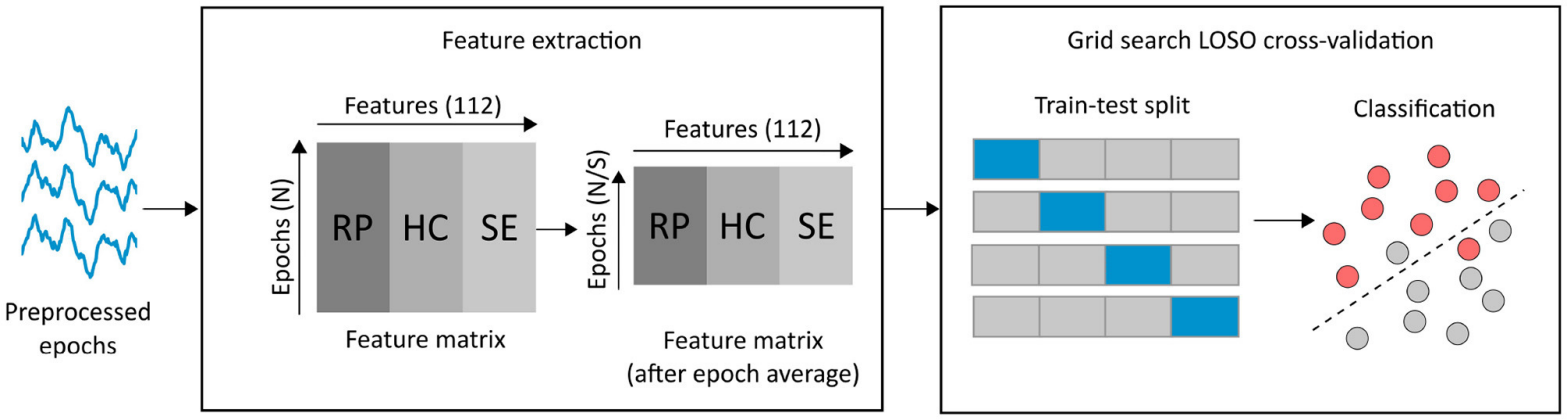
EEG-based feature extraction and classification pipeline. First, we performed feature extraction on the 4-s clean epochs from all the participants. This yielded a feature matrix with 112 columns (features) and as many columns as the total number of epochs (*N*). Next, we averaged every S consecutive epochs to enhance the SNR of the features (we evaluated values of 6, 8, 10, and 12 for S). Then, we implemented a grid search cross-validation procedure to find the best hyperparameters for two possible classifiers (SVM and LR). We selected a LOSO strategy for cross-validation. Finally, we estimated the classification performance metrics across all the participants.

To create the feature matrix, we vertically concatenated the features extracted for each participant. Consequently, we ended up with a feature matrix with 112 columns (features) and as many rows as the total number of epochs from all the participants (see Figure 3). Then, we averaged every S consecutive rows in the feature matrix following to the approach described in Fraga et al. (2013). We followed this approach with two objectives in mind: to enhance the signal to noise ratio of the features, and to reduce the size of the feature matrix. We evaluated four values for S: 6, 8, 10, and 12, and we reported the best for each classification problem (MCI-non-AD vs. NC and mild-AD vs. NC) in the Section 3. Therefore, for each classification problem, we utilized a feature matrix (RP, HC, and SE) and a target array holding the cohort corresponding to each row (epoch) in the feature matrix.

### 2.5. Classification

To solve the two binary classification problems examined in this study, we used scikit-learn Python module (Pedregosa et al., 2011) to implement a three-step classification pipeline including a feature scaler, a feature selector, and a classifier. The feature scaler normalizes each feature in the range between zero and one, in a way that those with higher order of magnitude are not favored during classification. Subsequently, the feature selector selects the most relevant features according to a particular strategy. In our case, we applied the chi-square test included as part of scikit-learn feature selection module to find the features most related to the target. We selected this method due to its intrinsic speed. We refer the interested reader to Chandrashekar and Sahin (2014) for a comprehensive overview of feature selection strategies. Finally, for the classifier, we evaluated two algorithms: SVM with a radial basis function kernel and logistic regression (LR). To find the best hyperparameters for the classification pipeline we implemented grid search. For the SVM, we explored different values for the regularization parameter (C) and the kernel coefficient (γ). The C parameter refers to the strength of the regularization that is applied to avoid overfitting. In particular, C influences the selection of the SVM hyperplane margin and its amplitude is inversely proportional to the regularization strength. On the other hand, the γ parameter defines the kernel function used by the SVM algorithm to define a notion of similarity between input data and transform the data in the feature space. Likewise, for the LR, we explored different values for the regularization parameter (C) and the weights associated with the classes. In the case of logistic regression, the regularization parameter controls a penalty term added to the loss function in order to avoid overfitting by penalizing extreme parameter weights. On the other hand, the class weight parameter allow the algorithm to associate different weights to the classes based on their support. We examined the default option using no class weights, and the balanced option that adjusts the weights inversely proportional to class frequencies. Figure 3 illustrates the pipeline described in this subsection.

To evaluate the generalization ability of the classifier, we performed cross-validation using a LOSO strategy. Under this strategy, data is split into as many folds as participants. For each fold, the training set includes data from all the participants but one, whose data is reserved for the test set. Hence, information from a participant is never in the training and the test set simultaneously, what prevents from positive bias. Furthermore, according to the comprehensive review by Cassani et al. (2018), this cross-validation strategy is the most extended across the studies that aim to classify AD groups from resting state EEG.

For the sake of clarity, it is important to note that, during cross-validation, the parameters corresponding to the three stages of the classification pipeline (feature scaling, feature selection, and classification) are estimated using the training set, and subsequently, those parameters are applied on the test set. This avoids overfitting and prevents from artificial positive bias.

## 3. Results

In this section, we present the results that we obtained for the two binary discrimination problems evaluated in this study: mild AD vs. NC and MCI-non-AD vs. NC, based on the 16-channel resting state EEG recordings that we acquired (see Section 2.2).

**Figure 4:**
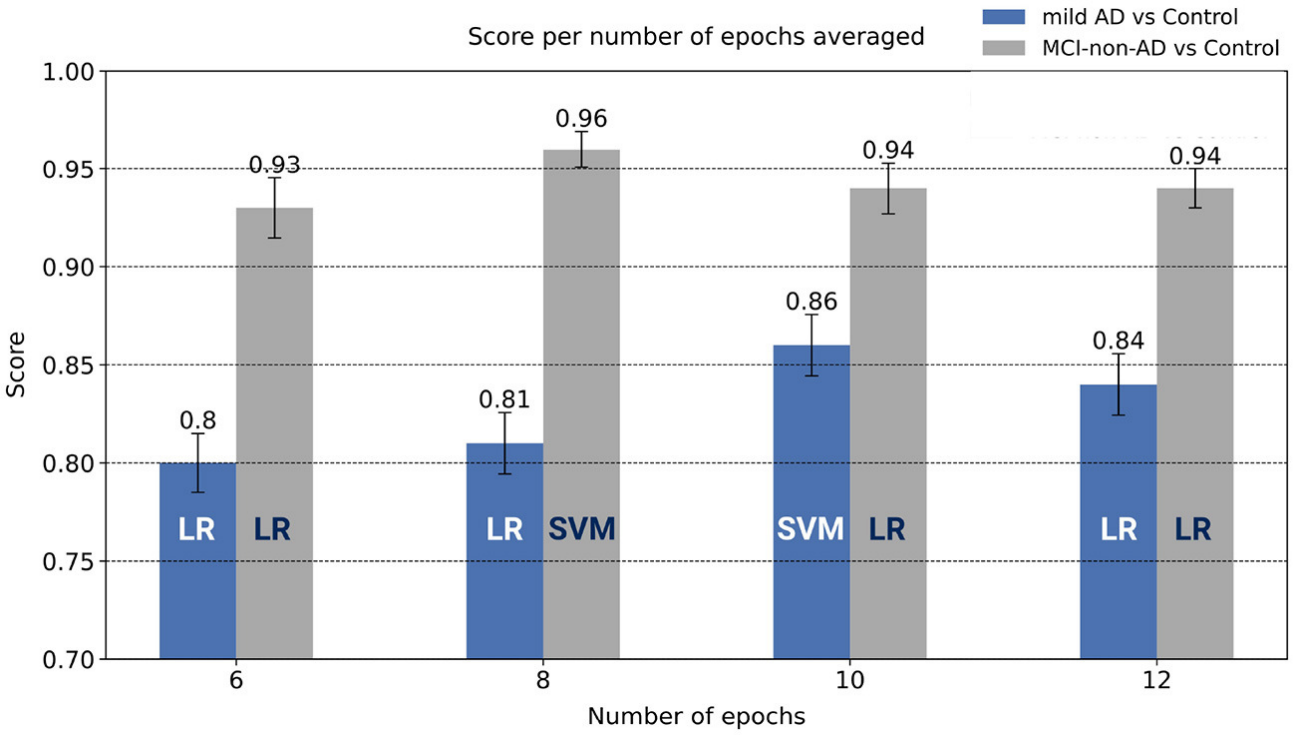
Comparison of the results yielded for the two binary classification problems as a function of the number of epochs averaged during processing. Error bars indicate the standard error of the mean. For each case, we have also included the name of the best performing classifier. For the mild AD vs. Control problem and the MCI-non-AD vs. Control problem, SVM with 10 and 8 epochs used for the average, yielded the best performance, respectively.

Figure 4 shows the average F1-score obtained during cross-validation as a function of the number of consecutive epochs averaged after feature extraction (see Section 2.4). To create this figure, we evaluated the classification pipeline on four different feature matrices (considering 6, 8, 10, and 12 consecutive epochs for the average).

Table 2 presents the hyperparameter values that we inspected through grid-search. We have also included the combination of hyperparameters that yielded the best cross-validation results. As shown in the right-most column of this table, the SVM classifier yielded the best performance for the two binary discrimination problems that we considered. Note that, in this table, we have not reported the best hyperparameters for the LR because the SVM outperformed this classifier on the two classification problems.

**Table 2:** Hyperparameter values inspected using grid-search cross-validation.

**Hyperparameter**	**Range**	**Best**	
		**Mild AD vs. NC**	**MCI-non-AD vs. NC**
% Features to keep	[10, 25, 50, 75, 100]	100	25
C (SVM)	[10^−4^, 10^−3^, …, 10^4^]	10^2^	10^1^
γ (SVM)	[Scale, auto]	auto	scale
C (LR)	[10^−4^, 10^−3^, …, 10^4^]	-	-
Class weight (LR)	[None, balanced]	-	-

“*Best” column indicates the values of each hyperparameter that resulted in the best performance. We have not reported the best values for the LR because the SVM algorithm yielded superior results*.

Table 3 holds the confusion matrices for the mild AD vs. NC and the MCI-non-AD vs. NC problems. As outlined in the previous paragraph, these results were yielded by the SVM classifier. The values outside the parenthesis represent the epoch-level confusion matrix, whilst the values inside the parenthesis represent the participant-level confusion matrix. To estimate the epoch-level matrix, we gathered the predictions and the true labels from all the cross-validation test sets, and we computed the percentage of correct predictions per class. Alternatively, for the participant-level matrix, we evaluated how well the classifier grouped each participant into one of the two groups for each binary classification problem. Consequently, if the classifier correctly guessed more than 50% of the epochs from a participant, we considered it correctly classified the participant; otherwise, we considered it missclassified the participant.

**Table 3:** Confusion matrices for the two binary classification problems.

		**Mild AD vs. NC**			**MCI-non-AD vs. NC**	
		PAT	HC		PAT	HC
PAT		0.88 (8)	0.12 (0)		0.88 (4)	0.12 (1)
HC		0.17 (1)	0.83 (7)		0.01 (0)	0.99 (8)

*They were estimated from the predictions yielded by the SVM, which was the best performing classifier for both problems. The values outside and inside the parenthesis represent the results for the epoch-level and the participant-level classification, respectively. The highlighted cells denote the true positives and true negatives*.

Table 4 shows the per-cohort cross-validation metrics yielded by the classification pipeline for the mild AD vs. NC and the MCI-non-AD vs. NC problems. We derived these metrics from the epoch-level confusion matrices described above.

**Table 4:** Classification metrics for the mild AD vs. NC and MCI-non-AD vs.

**Problem**	**Cohort**	**Precision**	**Recall**	**F1-score**
Mild AD vs. NC	Mild AD	0.83	0.88	0.86 ± 0.06
	NC	0.88	0.83	
MCI-non-AD vs. NC	MCI-non-AD	0.98	0.88	0.96 ± 0.03
	NC	0.92	0.99	

*NC problems. The right-most column indicates the average cross-validation F1-score ± the standard error of the mean*.

## 4. Discussion

The goal of this study was to perform a preliminary evaluation of an automated approach for the discrimination of AD cohorts. For this purpose, we recorded the EEG of a group of volunteers, and we proposed an approach based on a commercial EEG device with a reduced montage and an automated classification pipeline. The preliminary results that we obtained from a reduced sample suggest that such an approach can precisely discriminate mild AD and MCI-non-AD participants from healthy controls.

In Figure 4, we examined the epoch average procedure that we applied to the feature matrix. According to this figure, performance increased with the number of averaged epochs and then plateaued for both classification problems, what supports the application of the epoch average step described in Section 2.4. Regarding the classification problem of mild AD vs. NC, in view of the results presented in Table 3, our approach yielded promising results, both at the epoch and the participant levels. More specifically, according to the results presented in Table 4, recall for the mild AD cohort was the highest performance metric (0.88). This is particularly important because it evidences the capability of the classifier to detect the participants affected by the disease, what is crucial in studies that aim to discriminate clinical cohorts. Similarly, for the classification of MCI-non-AD vs. NC, the confusion matrices presented in Table 3 illustrate the sound performance of the classifier both at the epoch and the participant levels. According to the metrics reported in Table 4, the average performance of the classifier was higher for the MCI-non-AD vs. NC problem than for the mild AD vs. NC problem, as evidenced by average F1-scores of 0.96 and 0.86, respectively. An analogous study also reported better discrimination performance for the MCI vs. NC classification problem (Fiscon et al., 2018), although the reasons behind this result remain still unexplained. An evaluation of the presented approach on a larger sample size could contribute to elucidating this circumstance.

In terms of recording conditions, most studies in literature typically use between 17 and 32 electrodes (Cassani et al., 2018). However, to minimize participant distress, we selected only 16 electrodes. A similar number of electrodes was considered in related works (Wang et al., 2015; Kulkarni and Bairagi, 2017; Chen et al., 2018; Yu et al., 2020). With respect to epoch length, since EEG signals are non-stationary, we utilized an intermediate length of 4 s (Cassani et al., 2018), comparable to the duration selected in analogous works (Coronel et al., 2017; Mammone et al., 2017; Durongbhan et al., 2019). Regarding artifact rejection, works on AD detection traditionally have applied manual epoch selection for artifact removal (Simons et al., 2015; Azami et al., 2017; Mammone et al., 2017; Chen et al., 2018; Ruiz-Gomez et al., 2018), what may be counterproductive from the early detection standpoint. Alternatively, since we wanted to perform a preliminary evaluation of an automated classifier, we decided to perform artifact processing through Autoreject and automated ICA. The rationale behind the use of Autoreject was the interpolation of bad data spans supported by the algorithm, and its capability to perform data-driven rejection of artifactual segments. Regarding ICA, multiple works have used this analysis in a semi-automated way to identify and remove artifactual components (Vecchio et al., 2017). Alternatively, in other works, researchers implemented an automated version of ICA (Echtioui et al., 2020). We followed the latter approach to remove blink artifacts and comply with the automation constraints of our approach. Particularly, we identified the blink artifact component through correlation with the Fp1 channel time series. We selected this channel because it has the position that most resembles an electro-oculogram channel. With respect to cross-validation, the three main strategies adopted in literature are leave-one-out (Simons and Abasolo, 2017; Yang et al., 2019), k-fold (Durongbhan et al., 2019; Ieracitano et al., 2020), and leave-one-subject-out (Trambaiolli et al., 2017; Fiscon et al., 2018; Ruiz-Gomez et al., 2018). We selected LOSO because with this cross-validation strategy, data from a participant is not in the training and the test set simultaneously. This prevents the classifier from positive bias and thus, from rendering unrealistically good predictions. Lastly, in terms of performance, our results are comparable to similar works in literature (Kulkarni and Bairagi, 2017; Trambaiolli et al., 2017; Fiscon et al., 2018; Mazaheri et al., 2018), or even superior in some cases (Morabito et al., 2016; Cassani et al., 2017; Amezquita-Sanchez et al., 2019; Liu et al., 2020).

The preliminary results that we obtained hint at the potential of fully automated AD discrimination approaches. The use of wearable commercial EEG acquisition systems may enhance the accessibility of electrophysiology-based screening. Additionally, automated processing techniques such as those considered in the approach presented in this paper may contribute to reducing the personnel required for AD screening practice. Jointly, this could contribute to reducing the costs associated to AD screening and to speeding-up medical treatment. Consequently, we believe this preliminary study along with further research, may help to open the door for fast and portable EEG-based AD detection approaches that could integrate into the clinical ecosystem in the near future. Nonetheless, this preliminary study also presents some limitations. First, the sample size that we considered is too small to yield definitive conclusions. Therefore, future works must evaluate the presented approach on a larger sample to validate the conclusions drawn in this study. Furthermore, the participants that we recruited for this study do not represent the typical patient who attend the neurology services. Often, patients suffer from additional pathologies accompanied by symptoms that could overlap with those from dementia. Obviously, the inclusion of such participants would represent an important challenge for researchers, nonetheless, we believe the study of typical patients is crucial in order to potentially transfer these approaches into daily medical practice. Finally, although an automated AD detection approach could bring benefits in terms of economical costs and waiting times, it would also require the clinicians to be properly trained to use a commercial EEG acquisition system (wear the EEG headset, monitor channel impedance, and control the graphical user interface of the device, among other tasks).

## 5. Conclusions

In this paper, we preliminarily evaluated the feasibility of an automated pipeline based on EEG activity for the discrimination of mild AD and MCI-non-AD. For this purpose, we recorded the resting state activity of a reduced sample of volunteers using a commercial EEG device, and we designed our approach to automatically perform signal processing, feature extraction, and classification. We applied the Autoreject algorithm and ICA for artifact rejection. Then, we estimated the relative power, the Hjorth complexity, and the spectral entropy of the preprocessed epochs. Lastly, we assessed two binary classifiers (SVM and LR) for the discrimination of the three cohorts of interest *via* leave-one-subject-out cross-validation. The results that we obtained are comparable to the best reported in literature, what is promising toward the implementation of automated AD detection approaches based on commercial acquisition devices. Since we analyzed a reduced sample size, further studies must evaluate the presented approach on larger samples to validate the conclusions yielded in this paper. Nonetheless, the results obtained in this work are promising, as they suggest that AD detection could be performed automatically using a few minutes of resting state EEG activity and a commercial acquisition device. With this in mind, we believe the future inclusion of this kind of approaches into AD screening practice could contribute to reducing the costs linked to AD detection, and also enable the early detection of the disease, what could potentially advance medical treatments. Nonetheless, further research is also required to investigate the feasibility of automated EEG-based approaches in participants that suffer from additional pathologies whose symptoms overlap with those of dementia. This is often the case in daily-life neurological practice, hence, such cases must be comprehensively studied before future patients can take advantage from fast, accurate, and affordable detection techniques that enhance their standards of living.

## Data Availability Statement

The raw data supporting the conclusions of this article will be made available by the authors, without undue reservation.

## Ethics Statement

The studies involving human participants were reviewed and approved by Hospital Universitario Virgen de las Nieves de Granada. The patients/participants provided their written informed consent to participate in this study.

## Author Contributions

EP-V and ML-G: conceptualization and methodology. EP-V: software, formal analysis, investigation, resources, data curation, and writing—original draft preparation. EP-V, ML-G, and CM: validation. EP-V, ML-G, CM, RV-C, IC-M, and SL-A: visualization and writing—review and editing. ML-G and CM: supervision. ML-G, CM, RV-C, IC-M, and SL-A: project administration. All authors have read and agreed to the published version of the manuscript.

## Funding

This work was supported by project PID2021-128529OA-I00 Proyectos de Generación de Conocimiento 2021 funded by the Spanish Ministry of Science, Innovation and Universities, by European Regional Development Funds, and by project B-TIC-352-UGR20, and also co-funded by the Operative Program FEDER 2014–2020 and the Economy, Universities and Science Office of the Andalusian Regional Government.

## Conflict of Interest

The authors declare that the research was conducted in the absence of any commercial or financial relationships that could be construed as a potential conflict of interest.

## Publisher's Note

All claims expressed in this article are solely those of the authors and do not necessarily represent those of their affiliated organizations, or those of the publisher, the editors and the reviewers. Any product that may be evaluated in this article, or claim that may be made by its manufacturer, is not guaranteed or endorsed by the publisher.
